# Sociodemographic characteristics associated with hospital contact in the year prior to suicide: A data linkage cohort study in Victoria, Australia

**DOI:** 10.1371/journal.pone.0252682

**Published:** 2021-06-03

**Authors:** Angela Clapperton, Jeremy Dwyer, Ciara Millar, Penny Tolhurst, Janneke Berecki-Gisolf

**Affiliations:** 1 Melbourne School of Population and Global Health, University of Melbourne, Melbourne, Victoria, Australia; 2 Monash University Accident Research Centre, Monash University, Melbourne, Victoria, Australia; 3 Coroners Prevention Unit, Coroners Court of Victoria, Melbourne, Victoria, Australia; 4 Mental Health and Drugs Branch, Victorian Department of Health, Melbourne, Victoria, Australia; University of Sheffield, UNITED KINGDOM

## Abstract

**Aims:**

The aims of this study were to examine the prevalence of hospital contact in the year prior to suicide in Victoria, Australia, and to compare characteristics among those who did and did not have contact in the year prior to suicide.

**Methods:**

The study was a data linkage cohort study of 4348 Victorians who died by suicide over the period 2011–2017. Data from the Victorian Suicide Register (VSR) was linked with hospital separations and Emergency Department (ED) presentations datasets by the Centre for Victorian Data Linkages (CVDL). The main outcomes were: (1) hospital contact for any reason, (2) hospital contact for mental-health-related reasons, and (3) hospital contact for intentional self-harm. Unadjusted and adjusted odds ratios were calculated as the measures of association.

**Results:**

In the year prior to suicide, half of the decedents (50.0%) had hospital contact for any reason (n = 2172), 28.6% had mental-health-related hospital contact (n = 1244) and 9.9% had hospital contact for intentional self-harm (n = 432).

In the year prior to suicide, when compared with males aged 25–49 years (the reference group):

- males aged 75+ years and females of all ages were significantly more likely to have hospital contact for any reason
- females aged 10–24 years and 25–49 years were significantly more likely to have mental-health-related hospital contact
- females aged 10–24 years and 25–49 years had 3.5 times and 2.4 times the odds of having hospital contact for intentional self-harm.

**Conclusions:**

The comparatively high proportion of female decedents with mental-health related hospital contact in the year prior to suicide suggests improving the quality of care for those seeking help is an essential prevention initiative; this could be explored through programs such as the assertive outreach trials currently being implemented in Victoria and elsewhere in Australia. However, the sizeable proportion of males who do not have contact in the year prior to suicide was a consistent finding and represents a challenge for suicide prevention. Programs to identify males at risk in the community and engage them in the health care system are essential. In addition, promising universal and selective interventions to reduce suicide in the cohort who do not have hospital contact, include restricting access to lethal means and other public health interventions are also needed.

## Introduction

Many people who die by suicide have had contact with hospitals (i.e., outpatient or inpatient services) in the weeks or months leading up to their death [1–5]. These contacts represent potential suicide prevention opportunities either through identification of persons at risk of suicide, provision of treatment and/or delivery of interventions.

Consequently, across Australia and in many other jurisdictions, suicide prevention initiatives have been designed to target the population that access hospitals. For example, the Australian federal government has funded 12 National Suicide Prevention Trials involving primary health network (PHN) regions [6]. One of the interventions included in some of these trials aims to improve emergency and follow-up care for suicidal crisis by providing dedicated aftercare services for people who attempt suicide. In addition, one particular state government in Australia—the Victorian State Government–has a suicide prevention framework [7] that outlines two major initiatives for suicide prevention, one of which is the Hospital Outreach Post-suicidal engagement (HOPE) program. HOPE trials provide enhanced support and assertive outreach for people that present to the emergency departments for intentional self-harm.

By better understanding characteristics of people who have hospital contact prior to suicide, current strategies such as those mentioned here, can be further developed to improve access to services, encourage service engagement, and improve the quality of the services currently being provided. Conversely, by understanding who *does not* have contact with hospitals prior to suicide, other interventions can be developed specifically targeting those people who would not be helped by hospital outreach initiatives.

Three previous reviews were identified on the prevalence of contact with health care prior to suicide. The first review published in 1998 concluded that up to 41% of people who died by suicide had contact with inpatient psychiatric care in the year prior to their death [1]; however, this finding was based on just one study. The second review, published in 2002, found the proportion who had inpatient psychiatric contact ranged from 16% to 46% (average 32%) [3]. Both these reviews also found variation between age groups and genders in rates of contact. A more recent 2018 review found 26% of suicide decedents had contact with inpatient or outpatient mental health services in the year prior to suicide and that women had significantly higher levels of contact compared to men [8]. However, no Australian studies were included in this updated review, suggesting a lack of this kind of recent research in Australia overall and specifically in the state of Victoria. Importantly, prior research indicates most individuals are not seen in hospitals prior to suicide [1–5]; consequently, it is important to understand the characteristics of groups that do not present at hospital as well as those who do. Therefore, the aims of this study were to examine the prevalence of hospital contact in the year prior to suicide and to compare the characteristics of those who did and did not have hospital contact in the year prior to suicide.

## Methods

### Design

This study is a retrospective cohort study of 4348 Victorians who died by suicide over the period 2011–2017 and examines hospital contacts that occurred in the cohort in the 12-months prior to their death.

### Data sources

Suicide data from the Victorian Suicide Register (VSR) was linked with hospital separations from the Victorian Admitted Episodes Dataset (VAED) and Emergency Department (ED) presentations from the Victorian Emergency Minimum Dataset (VEMD). The Victorian Department of Health and Human Services (DHHS) was the source of the data from the VAED and VEMD. The VSR is an ongoing register established by the Coroners Court of Victoria (CCOV) to collate detailed information on suspected suicides to assist Coroners with their investigations and support their prevention mandate. All suspected suicides in Victoria are legally required to be reported to the Coroner for investigation [2]. The VSR has been found to have high sensitivity for capturing all suicides in comparison to national data [9]. Admitted patient activity is captured in the VAED; all Victorian public and private hospitals contribute to the VAED, allowing a comprehensive and complete population-based dataset. Cases recorded on the VAED are coded to ICD-10-AM: the WHO International Statistical Classification of Diseases and Related Health Problems, Tenth Revision, Australian Modification. The Victorian Emergency Minimum Dataset (VEMD) comprises demographic, administrative and clinical data detailing presentations at 38 Victorian public hospitals with designated 24-hour emergency departments. Health services without a 24-hour ED do not contribute data to the VEMD, so the collection covers the majority, but not necessarily all, of emergency presentations in the state of Victoria. Data are coded to the relevant VEMD User Manual published by DHHS [10]. The Coroners Court of Victoria (CCOV) supplied VSR data to the Centre for Victorian Data Linkage Unit (CVDL) via the secure data exchange (SDE) portal. The only VSR variables included were name, date of birth, age, sex and LGA of residence. CVDL linked the VSR variables with the included hospital data sets and all data were then de-identified. Only the de-identified datasets, containing a linkage ID, were transferred to researchers at Monash University using the SDE portal.

### Data selection and definitions

VSR data on all suspected and Coroner-determined suicides was supplied for the period 1 January 2011 to 31 December 2017. In hospital datasets, contacts were limited to those that occurred in the year prior to suicide using the date of death recorded in the VSR and dates of contact provided in admissions and ED presentations datasets. Hospital admissions with an admission source indicating a transfer from another hospital or statistical separation (change in care type within the same hospital) in consecutive records were all considered to be part of the same episode and recoded to be the one hospital admission. Any hospital episode (ED presentation, hospital admission) that ended in “death” was excluded as it was considered to be a result of the fatal (suicide) incident. The only exception to this were episodes where the intentional self-harm was coded as occurring in a health service area–these episodes were retained as they were most likely inpatient suicides.

A hospital contact was considered to be mental health (MH) related if it was for reasons related to mental disorder, intentional self-harm and/or suicidal ideation. Hospital admissions were flagged as MH related if any of the 40 diagnosis codes were mental disorder (F00-F99), intentional self-harm (ISH) (X60-X84) or suicidal ideation (R45.81). ED presentations were flagged as MH related if any diagnosis code was mental disorder (F00-F99) or suicidal ideation (R45.81) or if the “human intent” variable indicated ISH. Records with a primary diagnosis code for a general psychiatric examination (Z046) or a history of ISH (Z915) were also included in the definition of MH related ED presentations.

The area-level measure of socioeconomic circumstances was taken from the Socio Economic Index For Areas (SEIFA) [11]. The Index of Relative Socio-economic Advantage and Disadvantage (IRSAD), which provides information about the economic and social conditions of people was used. A low score indicates relatively greater disadvantage and a lack of advantage in general whereas, a high score indicates a relative lack of disadvantage and greater advantage in general. As the SEIFA index is provided as a rank, we transformed it into quintiles, and it was applied at the Local Government Area (LGA) level by place of residence of the deceased.

### Statistical analysis

Descriptive analysis was carried out to identify sociodemographic characteristics of those who had hospital contact and those who did not. Chi square tests were conducted on categorical data to establish differences between the groups. The outcome variables were hospital contact yes/no, MH related hospital contact yes/no and ISH hospital contact yes/no).

Statistical modelling was carried out to determine sociodemographic factors associated with hospital contact. Univariate and multivariate logistic regression analysis including main effects of all variables was conducted using IBM SPSS version 26. Unadjusted and adjusted odds ratios were calculated as the measures of association. Due to a significant interaction between age and sex, these variables were combined and categorised into m10-25, m25-49, m50-74, m75+, f10-25, f25-49, f50-74, f75+.

### Ethics approval

This study was approved by the Monash University Human Research Ethics Committee; application number 14647. Consent was waived by the ethics committee as data was either collected for administrative purposes (hospital data) or participants were deceased (VSR data). All datasets were fully anonymised before being supplied to university researchers.

## Results

The cohort comprised 4,348 individuals who died by suicide over the period 2011–2017 and was 74.4% male. Half of the decedents (50.0%) had **hospital contact (HC) for any reason** during the year prior to suicide (n = 2172). Female decedents were significantly more likely than male decedents to have HC. This pattern was observed overall and in every age group with the exception of 75+ years ([overall 56.8% v 47.6%, p < .0001]; [10–24 years 60.4% v 44.7%, p = .001]; [25–49 years 56.7% v 47.6%, p < .0001]; [50–74 years 54.4% v 45.5%, p = .004]) (Table 1).

**Table 1:** Demographic characteristics associated with hospital contact (n = 4348).

	Any hospital contact						
	YES (n = 2172)		NO (n = 2176)		ALL	Unadjusted odds ratios (95% CI; p-value)	Adjusted odds ratios (95% CI; p-value)
	n	%	n	%	n		
**SEX & AGE**							
male 10–24	180	44.7	223	55.3	403	0.9 (0.7–1.1; p = .29)	0.9 (0.7–1.1; p = .26)
male 25–49	750	47.6	825	52.4	1575	1.0	1.0
male 50–74	451	45.5	540	54.5	991	0.9 (0.8–1.1; p = .30)	0.9 (0.8–1.1; p = .34)
male 75+	159	59.6	108	40.4	267	1.6 (1.2–2.1; p < .001)*	1.6 (1.2–2.1; p < .001)*
All male	1540	47.6	1696	52.4	3236		
female 10–24	93	60.4	61	39.6	154	1.7 (1.2–2.4; p = .003)*	1.7 (1.2–2.4; p = .002)*
female 25–49	282	56.7	215	43.3	497	1.4 (1.2–1.8; p < .001)*	1.5 (1.2–1.8; p < .001)*
female 50–74	202	54.4	169	45.6	371	1.3 (1.0–1.7; p = .02)*	1.3 (1.0–1.7; p = .02)*
female 75+	55	61.1	35	38.9	90	1.7 (1.1–2.7; p = .014)*	1.7 (1.1–2.7; p = .02)*
All female	632	56.8	480	43.2	1112		
**REGIONALITY**							
Metropolitan	1445	49.9	1449	50.1	2894	1.0	1.0
Rural/regional	727	50.0	727	50.0	1454	1.0 (0.9–1.1; p = .97)	1.0 (0.8–1.1; p = .70)
**SEIFA**							
IRSAD 1 (greatest disadvantage & lowest advantage)	296	52.9	264	47.1	560	1.0	1.0
IRSAD 2	318	53.3	279	46.7	597	1.0 (0.8–1.3; p = .89)	1.0 (0.8–1.3; p = .75)
IRSAD 3	366	49.1	380	50.9	746	0.9 (0.7–1.1; p = .18)	0.9 (0.7–1.1; p = .21)
IRSAD 4	609	48.7	642	51.3	1251	0.8 (0.7–1; p = .10)	0.8 (0.7–1.0; p = .10)
IRSAD 5 (lowest disadvantage & greatest advantage)	553	50.0	554	50.0	1107	0.9 (0.7–1.1; p = .26)	0.9 (0.7–1.1; p = .16)

Note: OR are calculated based on 4261 cases as 87 cases had missing or unspecified LGA of residence recorded on the VSR.

More than one-quarter of the suicide cohort (28.6%) had **MH HC** during the year prior to suicide (n = 1244). Female decedents were significantly more likely than male decedents to have MH HC in the year prior to suicide. This pattern was observed overall and and in every age group ([overall 36.4% v 25.9%, p < .0001]; [10–24 years 40.9% v 25.3%, p < .0001]; [25–49 years 40.0% v 30.3%, p < .0001]; [50–74 years 32.3% v 22.4%, p < .0001]; [75+ years 25.6% v 13.9%, p = .016]) (Table 2).

**Table 2:** Demographic characteristics associated with mental health related hospital contact (n = 4348).

	Any mental-health hospital contact						
	YES (n = 1244)		NO (n = 3104)		ALL	Unadjusted odds ratios (95% CI; p-value)	Adjusted odds ratios (95% CI; p-value)
	n	%	n	%	n		
**SEX & AGE**							
male 10–24	102	25.3	301	74.7	403	0.8 (0.6–1.0; p = .05)*	0.8 (0.6–1.0; p = .07)
male 25–49	478	30.3	1097	69.7	1575	1.0	1.0
male 50–74	222	22.4	769	77.6	991	0.7 (0.6–0.8; p < .001)*	0.7 (0.6–0.8; p < .001)*
male 75+	37	13.9	230	86.1	267	0.4 (0.3–0.5; p < .001)*	0.4 (0.3–0.5; p < .001)*
All male	839	25.9	2397	74.1	3236		
female 10–24	63	40.9	91	59.1	154	1.6 (1.1–2.2; p = .01)*	1.6 (1.1–2.2; p = .007)*
female 25–49	199	40.0	298	60.0	497	1.5 (1.2–1.9; p < .001)*	1.5 (1.2–1.9; p < .001)*
female 50–74	120	32.3	251	67.7	371	1.1 (0.9–1.4; p = .45)	1.1 (0.9–1.4; p = .41)
female 75+	23	25.6	67	74.4	90	0.8 (0.5–1.3; p = .34)	0.8 (0.5–1.2; p = .27)
All female	405	36.4	707	63.6	1112		
**REGIONALITY**							
Metropolitan	875	30.2	2019	69.8	2894	1.0	1.0
Rural/regional	369	25.4	1085	74.6	1454	0.8 (0.7–0.9; p < .001)*	0.7 (0.6–0.9; p < .001)*
**SEIFA**							
IRSAD 1 (greatest disadvantage & lowest advantage)	142	25.4	418	74.6	560	1.0	1.0
IRSAD 2	173	29.0	424	71.0	597	1.2 (0.9–1.6; p = .17)	1.3 (1.0–1.7; p = .08)
IRSAD 3	229	30.7	517	69.3	746	1.3 (1.0–1.7; p = .03)*	1.2 (1.0–1.6; p = .01)
IRSAD 4	344	27.5	907	72.5	1251	1.1 (0.9–1.4; p = .34)	0.9 (0.7–1.2; p = .40)
IRSAD 5 (lowest disadvantage & greatest advantage)	335	30.3	772	69.7	1107	1.3 (1.0–1.6; p = .04)*	1.0 (0.8–1.3; p = .82)

Note: OR are calculated based on 4261 cases as 87 cases had missing or unspecified LGA of residence recorded on the VSR.

Ten percent of the suicide cohort (9.9%, n = 432 individuals) had **HC for ISH** during the year prior to suicide and female decedents were significantly more likely than male decedents to have ISH HC. This pattern was observed overall and among those aged 10–24 years and 25–49 years ([overall 15.9% v 7.9%, p < .0001]; [10–24 years 24.0% v 9.7%, p < .0001]; [25–49 years 18.5% v 8.4%, p < .0001]) (Table 3).

**Table 3:** Demographic characteristics associated with hospital contact for intentional self-harm (n = 4348).

	Any hospital contact for intentional self-harm						
	YES (n = 432)		NO (n = 3916)		ALL	Unadjusted odds ratios (95% CI; p-value)	Adjusted odds ratios (95% CI; p-value)
	n	%	n	%	n		
**SEX & AGE**							
male 10–24	39	9.7	364	90.3	403	1.2 (0.8–1.7; p = .41)	1.2 (0.8–1.7; p = .36)
male 25–49	132	8.4	1443	91.6	1575	1.0	1.0
male 50–74	74	7.5	917	92.5	991	0.9 (0.7–1.2; p = .41)	0.9 (0.7–1.2; p = .53)
male 75+	10	3.7	257	96.3	267	0.4 (0.2–0.8; p = .011)*	0.4 (0.2–0.9; p = .02)*
All male	255	7.9	2981	92.1	3236		
female 10–24	37	24.0	117	76.0	154	3.5 (2.3–5.2; p < .001)*	3.5 (2.3–5.3; p < .001)*
female 25–49	92	18.5	405	81.5	497	2.5 (1.9–3.3; p < .001)*	2.4 (1.8–3.2; p < .001)*
female 50–74	40	10.8	331	89.2	371	1.3 (0.9–1.9; p = .14)	1.3 (0.9–2.0; p = .12)
female 75+	8	8.9	82	91.1	90	1.1 (0.5–2.3; p = .87)	1.0 (0.5–2.2; p = .95)
All female	177	15.9	935	84.1	1112		
**REGIONALITY**							
Metropolitan	316	10.9	2578	89.1	2894	1.0	1.0
Rural/regional	116	8.0	1338	92.0	1454	0.7 (0.6–0.9; p = .002)*	0.7 (0.5–0.9; p = .02)*
**SEIFA**							
IRSAD 1 (greatest disadvantage & lowest advantage)	39	7.0	521	93.0	560	1.0	1.0
IRSAD 2	57	9.5	540	90.5	597	1.4 (0.9–2.2;p = .11)	1.6 (1.0–2.4; p = .05)*
IRSAD 3	80	10.7	666	89.3	746	1.6 (1.1–2.4;p = .02)*	1.6 (1.1–2.4; p = .03)*
IRSAD 4	122	9.8	1129	90.2	1251	1.4 (1.0–2.1;p = .06)	1.2 (0.8–1.8; p = .41)
IRSAD 5 (lowest disadvantage & greatest advantage)	125	11.3	982	88.7	1107	1.7 (1.2–2.5;p = .006)*	1.4 (0.9–2.1; p = .13)

Note: OR are calculated based on 4261 cases as 87 cases had missing or unspecified LGA of residence recorded on the VSR.

### Multivariate analysis

Compared with males aged 25–49 years, males aged 75+ years had 1.6 times the odds of having HC, and females of all ages were significantly more likely to have HC (10–24 years 1.7x; 25–49 years 1.5x; 50–74 years 1.3x; 75+ years 1.7x) (Table 1).

For MH HC prior to suicide, when compared with males aged 25–49 years, older males aged 50–74 years and 75+ had reduced odds of MH HC (0.7x and 0.4x respectively), whereas females aged 10–24 years and 25–49 years were more likely to have MH HC (1.6x and 1.5x respectively). Rural/regional residents had lower odds of having MH HC when compared to metropolitan residents (0.7x) (Table 2).

For ISH HC, females aged 10–24 years and 25–49 years had 3.5 times and 2.4 times the odds of having ISH HC when compared to males aged 25–49 years. The oldest males (75+ years) had reduced odds of ISH HC (0.4x). Rural/regional residents had lower odds of having ISH HC when compared to metropolitan residents (0.7x) and persons in SEIFA quintiles 2 & 3 were more likely than persons in quintile 1 to have ISH HC (1.6x) (Table 3).

#### Groups with the lowest proportion of individuals having HC prior to suicide

There were many groups that had very low proportions of decedents who had HC in the year prior to suicide (Table 4). With respect to HC for any reason, most of the groups with the least contact were aged 10–49 years, regional/rural residents and were males. When contact for MH was examined, all groups with the lowest proportion of contact were male and many were aged 50+ years. In eight groups none of the decedents had hospital contact in the year prior to suicide for ISH–all were male, all age groups were represented as were regional/rural residents and metropolitan residents.

**Table 4:** Ten groups with the lowest proportion of decedents having hospital contact, mental-health contact and intentional self-harm contact in the year prior to suicide.

Any contact			Any mental-health related contact			Intentional self-harm related contact		
Group	Tot. n	% no contact	Group	Tot. n	% no contact	Group	Tot. n	% no contact
females 25–49, R, 4	12	66.7	males 75+, R, 1	31	93.5	males 75+, M, 1	15	100.0
males 25–49, R, 5	15	66.7	males 75+, M, 1	18	88.9	males 75+, M, 3	20	100.0
males 25–49, R, 4	32	65.6	males 75+, M, 5	67	88.1	males 75+, R, 1	15	100.0
males 10–24, M, 2	13	61.5	males 50–74, R, 4	23	87.0	males 50–74, R, 5	31	100.0
males 50–74, R, 4	23	60.9	males 75+, R, 2	30	86.7	males 25–49, R, 4	33	100.0
males 10–24, M, 5	87	59.8	males 10–24, M, 2	13	84.6	males 25–49, R, 5	32	100.0
females 25–49, M, 1	24	58.3	males 75+, R, 3	28	82.1	males 10–24, M, 1	21	100.0
males 50–74, R, 3	96	58.3	males 10–24, R, 3	33	81.8	males 10–24, R, 3	18	100.0
males 10–24, R, 2	43	58.1	males 50–74, M, 1	44	81.8	males 50–74, M, 1	44	97.7
males 10–24, R, 3	33	57.6	males 50–74, M, 4	244	81.6	males 75+, M, 5	67	97.0

R = regional/rural, M = metropolitan; numbers 1–5 represent the IRSAD quintiles: 1 = greatest disadvantage & lowest advantage—5 = lowest disadvantage & greatest advantage.

Note: OR are calculated based on 4261 cases as 87 cases had missing or unspecified LGA of residence recorded on the VSR.

## Discussion

This is the first study that has linked Victorian Suicide Register (VSR) data with hospital administrative datasets to gain insight into the incidence of hospital contact in the year prior to suicide. Previous work using the VSR has examined service contacts and/or history of previous mental-health-related issues but used VSR coded data in isolation potentially raising issues of accuracy and completeness [2,12–14].

Half of the 4348 decedents had hospital contact for any reason in the year prior to suicide, 29% had contact for mental health related reasons and 10% had contact for intentional self-harm. These results regarding mental health contacts are consistent with a 2018 review that found 26% of suicide decedents had contact with inpatient or outpatient mental health services in the year prior to suicide [8]. The intentional self-harm results are consistent with a 2016 US study reporting that among cases who died by suicide, 10% had an external cause of injury code for self-inflicted injury on their ED record in the six week period prior to suicide [15].

Although almost three-quarters of the suicide cohort were male (74%), females were significantly over-represented in hospital-treated contacts for most age groups. This may simply reflect difference in the prevalence of help seeking by males and females in the general population but may also provide valuable suicide intervention and prevention guidance. The comparatively high proportion of female decedents (particularly young females) with mental-health related hospital contact in the year prior to suicide suggests improving the quality of care for women who are seeking help is an essential suicide prevention initiative; this could be explored through the assertive outreach trials currently being implemented in Victoria and elsewhere in Australia. The sizeable proportion of men who do not have hospital contact in the year prior to suicide was a consistent finding across age groups, regions and SEIFA quintiles, and represents a particular challenge. Programs to identify males at risk in the community and engage them in the health care system are essential. In addition, promising universal and selective interventions to reduce suicide in the cohort who do not have hospital contact, include restricting access to lethal means [16–18] and other public health interventions are also needed. One such promising intervention includes a three-part documentary called Man Up which was found to significantly increase males’ intentions to seek help for personal and emotional problems [19].

Only 13.9% of men and 25.6% of women decedents aged 75 years and older had mental-health-related contact recorded in the year prior to suicide; these numbers reduce to 3.7% and 8.9%, respectively, when contact for intentional self-harm is examined. These findings could suggest that suicide among older people in the Victorian population may be related to physical illness and are consistent with previous international [20] and Victorian [21] research that found physical illness was associated with increased suicide risk among older people. Health professionals in contact with older people who are experiencing serious physical illness should be encouraged to enquire about mental-health-related issues, including suicidal ideation, and when necessary, direct relevant patients to appropriate services. However, these findings could also be related to a lack of help-seeking, referral, or treatment for mental health issues among older people. Suicide prevention in this population clearly requires a unique approach, in terms of content as well as delivery. Considering our findings that most older people who died by suicide were not in contact with hospitals for their mental health, it is promising that a recent review examining the effectiveness of interventions to prevent suicidal ideation and behaviours in this population, found the most beneficial results were associated with depression screening and management programs in the primary care setting [22]. Further, given this effectiveness of depression screening and management programs, it is suggested that such interventions in the general hospital setting may therefore be worthy of greater attention and research for the population who are in contact with hospitals.

### Limitations

As is common to all data linkage studies using administrative data, the information captured is limited to what was recorded in the administrative data. The quality and coding issues inherent in the different hospital data sources can influence the utility of the data for informing suicide prevention initiatives. Information about emergency presentations at health services without a 24-hour ED are not available in the VEMD, so the study covers the majority, but not necessarily all, of the emergency presentations among the cohort. Another data issue relates to known data quality issues with the VEMD regarding the coding of the “human intent” variable [23]. Of particular relevance to this study is that the social sensitivity that surrounds intentional self-harm and uncertainty regarding intent can lead to underestimates of self-harm incidents in hospital-treated datasets [23]. Consequently, incomplete coding of human intent (i.e. missing data) has likely led to an underestimate of the proportion of the cohort coded as having intentional self-harm related hospital contacts in the year prior to suicide.

### Conclusion

The findings of this research suggest that interventions at the universal, selective and indicated levels are all needed to prevent suicide and provide support for researchers that have argued for public health responses, interventions and upstream prevention to decrease suicide [24–27]. A common suicide prevention approach has been indicated interventions, which aim to identify and treat individuals who are self-harming, experiencing suicidal ideation or who are experiencing symptoms and disorders that have been linked to suicide–particularly targeting people treated in hospitals. These kinds of interventions are clearly necessary and should be encouraged due to their potential to help prevent suicide in the 29% of Victorian suicide decedents who have mental-health-related hospital contacts in the year prior to suicide. However, universal and selective interventions could benefit those at risk for suicide regardless of whether they have contact with hospitals.
